# Molecular Cloning and Characterization of Three Glucosinolate Transporter (GTR) Genes from Chinese Kale

**DOI:** 10.3390/genes10030202

**Published:** 2019-03-08

**Authors:** Ding Jiang, Jianjun Lei, Bihao Cao, Siyuan Wu, Guoju Chen, Changming Chen

**Affiliations:** 1 Key Laboratory of Biology and Germplasm Enhancement of Horticultural Crops in South China, Ministry of Agriculture, South China Agricultural University, Guangzhou 510642, China; jiangding92@gmail.com (D.J.); jjlei@scau.edu.cn (J.L.); caobh01@scau.edu.cn (B.C.); wsy-yuan@stu.scau.edu.cn (S.W.); 2 Guangdong Vegetable Engineering and Technology Research Center, College of Horticulture, South China Agricultural University, Guangzhou 510642, China

**Keywords:** Chinese kale, glucosinolate transport, expression analysis, gene function analysis

## Abstract

Chinese kale is a native vegetable in Southern China and the flowering stalk is the most commonly used edible part due to its high glucosinolate content and other nutritional qualities. The GTR protein played important roles in the glucosinolate transport process. In this study, three BocGTR1 genes were cloned from Chinese kale for the first time. Their gene structure, physicochemical properties, signal peptides, transmembrane structures, functional domains, second and third-order protein structures, and phylogenetic relationships were predicted. The expression levels of BocGTR1a and BocGTR1c were much higher than those of BocGTR1b in various tissues, especially in leaves and buds. In addition, the expression patterns of three genes were examined under various abiotic stresses or hormone treatment, including those induced by wounding, heat stress, methyl jasmonate, salicylic acid, salt, and MgCl_2_ treatment. BocGTR1a and BocGTR1c were strongly induced by wounding and heat stress. The expression of BocGTR1a and BocGTR1c was significantly silenced in plants transformed by RNAi technology. Total glucosinolate content was significantly decreased in mature leaves and increased in roots of RNAi-transformed plants compared to wild-type plants. In addition, we found that BocGTR1a and BocGTR1c may participate in glucosinolate accumulation in different tissues with a selection for specific glucosinolates. These results indicated that BocGTR1a and BocGTR1c may be the key genes involved in the glucosinolate accumulation in different tissues of Chinese kale.

## 1. Introduction

Glucosinolates are a group of secondary metabolites containing nitrogen and sulfur, mainly found in the order Capparales. These metabolites play important roles in plant defence and in human nutrition [1,2]. It has been clinically proven that some glucosinolate-derived isothiocyanate and nitrile compounds display anticarcinogenic activity [1,2]. Depending on the amino acids from which they are synthesized, glucosinolates are divided into three major groups: Aliphatic, indolyl, and aromatic glucosinolates [2]. Generally, the biosynthesis of glucosinolate occurs via three separate phases: The chain elongation of precursor amino acids, the formation of the core structure, and modifications of the side chain of glucosinolate [3]. A number of key regulators and genes involved in the biosynthetic network of glucosinolate that are present in the genus *Arabidopsis* are known [3,4]. Glucosinolates are synthesized in every part of plants except seeds and are then transported to storage positions in seeds and other parts. Glucosinolates were synthesized in other plant parts and eventually accumulate in seeds by GTR protein transport [5]. However, the molecular mechanisms of the GTR-dependent transport system for regulating the accumulation level of different types of glucosinolate in *Brassica* vegetables are still unknown.

Initially, researchers predicted that secondary metabolites often migrated in plants [6]. Therefore, many researchers have tried to explore whether glucosinolates truly transfers and how this transfer occurs. Lykkesfeldt and Møller [7] studied the biosynthesis of benzyl glucosinolate in various organs during the development of *Trollius chinensis* with radioisotope C14. The results showed that benzyl glucosinolate was mainly synthesized in leaves, but accumulated in seeds and other tissues. The amount of glucosinolates in seeds was measured in 39 ecotypes of *Arabidopsis thaliana*, showing a positive correlation between the content of aliphatic glucosinolates in leaves and seeds, which suggested that glucosinolates are transferred from leaves to seeds [8]. The content of glucosinolates in plant seeds is higher than that in other tissues and organs, but the amount of glucosinolates synthesized in seeds is very small. Therefore, there is a transporting system for glucosinolates in plants [9,10].

The transporter genes ATGTR1 (AT3G47960) and ATGTR2 (AT5G62680) were identified in *Arabidopsis thaliana* [5]. GTR transporters play an important role in the transportation of glucosinolates in plants. There was no accumulation of glucosinolate in the seeds of GTR1 and GTR2 mutants, while the content of glucosinolate in wild-type seeds was very high. The content of glucosinolate in the leaves of double mutants was more than 10 times higher than that in wild-type leaves [5]. Translocation and accumulation of glucosinolates in single-gene mutants of AtGTR1 or AtGTR2 were also affected, but the effect was significantly lower than that in double mutants, suggesting that if one of the GTR proteins were inhibited, the other would compensate for the loss of the GTR protein [5]. GTR proteins participate in long-distance two-way transport of aliphatic glucosinolates between leaves and roots through phloem and xylem, but cannot transport indole glucosinolates, which indicates that GTR proteins may specifically transport a certain class of glucosides [11]. Long-chain aliphatic glucosinolates are synthesized and accumulated in both roots and rosette leaves, but the root is the main storage site. Plants can regulate the composition and content of glucosinolates in roots and rosette leaves through a GTR-dependent glucosinolate transport system [11].

To identify the role of GTR protein in regulating glucosinolate accumulation in *Brassica* crops, one out of seven and four out of twelve GTR orthologs were knocked out and they reduced glucosinolate levels in seeds by 60%–70% in *B. rapa* and *B. juncea* [12]. This indicates that there are many GTR homologous proteins in *Brassica rapa* and *Brassica juncea*, which maintain glucosinolate transport function together, and there are key GTR proteins controlling glucosinolate transport to seeds for storage. Short-chain aliphatic glucosinolates are the main glucosinolates in Chinese kale, and the content of short-chain aliphatic glucosinolate in the root is higher than that in leaves and flowers, indicating that the root may be the storage site of short-chain aliphatic glucosinolate [13]. Therefore, the molecular mechanism of the glucosinolate transport system in Chinese kale may be different from that in *Arabidopsis*.

Chinese kale (*Brassica oleracea* var. *chinensis* Lei) is a native Chinese brassica vegetable that is widely distributed in Southern China and Southeast Asia [14]. In addition to good flavor, the flower stalk and leaves are high in anticarcinogenic compounds and antioxidants, including vitamin C, total phenolics, carotenoids, and glucosinolates [13,15,16,17,18]. Generally, the tender leaves and flowering stalks are the most common edible parts of Chinese kale [14]. To regulate the accumulation level of glucosinolate in different parts, it is very important to understand the molecular mechanism of glucosinolate transport in Chinese kale. In addition, many previous studies reported that some stress treatment or environmental factors, including radiation, temperature, light, CO_2_ concentration, and salinity, could affect the accumulation of glucosinolates in plants [19]. Therefore, it is very important to study the molecular mechanism of accumulation of glucosinolates under different stress conditions. In the present study, to identify the key genes involved in glucosinolate transport and re-accumulation in different tissues, three BocGTR1 genes were cloned from Chinese kale. The gene structure, physicochemical properties, signal peptides, functional domains, and phylogenetic relationships of three BocGTR1s were analyzed. The expression patterns of three genes were analyzed in various tissues and under different treatments and stresses. The function of BocGTR1a in Chinese kale was preliminarily analyzed by RNAi technology.

## 2. Results

### 2.1. Molecular Cloning of Three BocGTR1s

**Figure 1:**
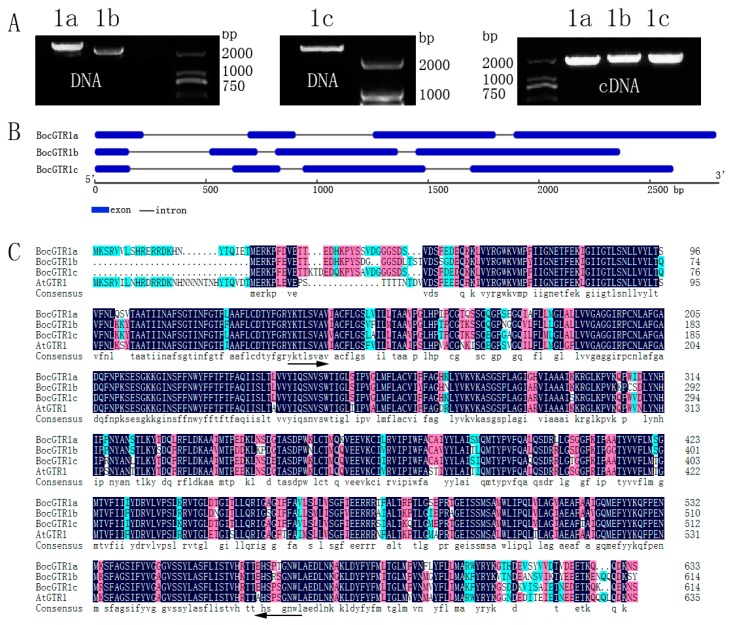
The full-length DNA, cDNA and deduced amino acid sequence of BocGTR1s. (**A**) The results of the full-length cDNA and DNA of three BocGTR1s. 1a, BocGTR1a; 1b, BocGTR1b; and 1c, BocGTR1c. (**B**) A schematic representation of the exon and intron organization of three BocGTR1s. BocGTR1s consist of 4 exons (blue boxes) and 3 introns (intervening line). (**C**) Alignment of the deduced amino acid sequence of three BocGTR1s from Chinese kale with AtGTR1 from *Arabidopsis thaliana*. The region of arrows is the conserved domain of PTR2 (Pfam: PF00854). The identical amino acids are marked in the dark background, 75% conserved amino acids in pink, and 50% conserved amino acids in green.

To isolate the homologous GTR genes in Chinese kale, we searched the *Brassica oleracea* genomic database [20] using the cDNA of AtGTR1(AT3G47960) as a probe sequence and three *Brassica oleracea* (*B. oleracea* var. *capitata*) GTR homology genes Bo3g113800.1, Bo1g076800.1, and Bo3g137030.1 were obtained. Chinese kale (*Brassica oleracea* var. *chinensis* Lei) belong to *Brassica oleracea*. Therefore, specific primers were designed based on the sequence of the three *B. oleracea* GTR1 homology genes above. The PCR amplification of the target fragment was performed using the DNA and cDNA of Chinese kale (Zhonghua) as templates, and three GTR1 were obtained, which were named *BocGTR1a*, *BocGTR1b*, and *BocGTR1c*, respectively (Figure 1A).

Agarose gel electrophoresis showed that the PCR fragment of DNA was longer than cDNA (Figure 1A), which indicated that these three BocGTR1s have introns. NCBI BLAST was used for retrieval and alignment of the nucleotide sequence. The DNA sequences of *BocGTR1a,b* and *c* were 2798 bp, 2365 bp and 2605 bp, respectively, and the full-length cDNAs of *BocGTR1a,b* and *c* were 1905 bp, 1848 bp and 1848 bp, respectively. The structure of the BocGTR1s encoded protein was analyzed by GSDS2.0 software. These three genes have the same gene structure number (3 introns and 4 exons), but the length of introns differed among the genes (Figure 1B). The structure of the encoded protein of the three BocGTR1s have a similarity of >99% with the corresponding homologous gene, Bo3g113800.1, Bo1g076800.1, and Bo3g137030.1. *BocGTR1a,b,c* have similarities of 83%, 81%, and 85% with AtGTR1 (At3G47960), respectively. In addition, the similarity among the three BocGTR1s was 88.18%, as analyzed by Soft DNAMAN 7.0 (Figure 1C), which indicated that these three genes belonged to the same gene family as *AtGTR1*.

### 2.2. Bioinformatics Analysis of Three BocGTR1s

**Figure 2:**
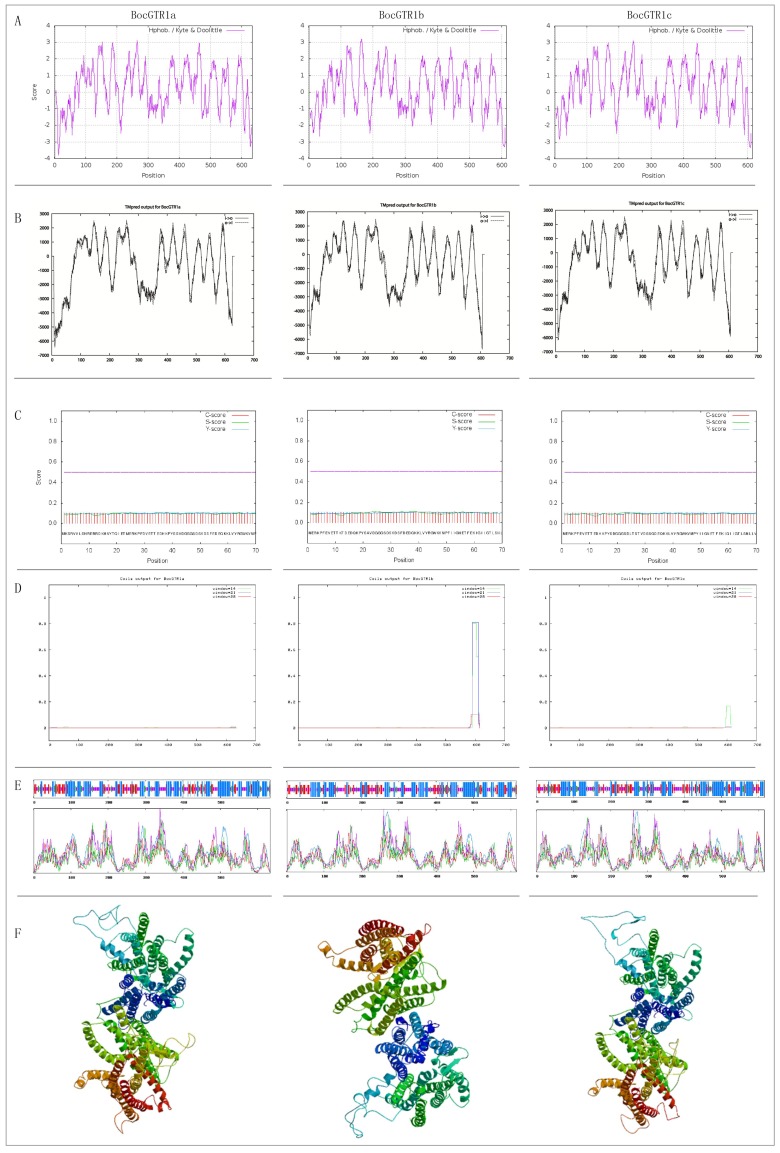
Bioinformatics analysis of the deduced amino acids of three BocGTR1s. (**A**) Prediction of hydrophobicity of BocGTR1s protein using ProtScale server. (**B**) Prediction of the trans-membrane structure of the BocGTR1s protein using TMpred server. (**C**) Prediction of the BocGTR1s protein signal peptide using SignalP server. (**D**) Prediction of the BocGTR1s protein crimp helix using COILS server. (**E**) Prediction of the two-dimensional structure of BocGTR1s using SOPMA server. (**F**) Prediction of the three-dimensional structure of BocGTR1s using the SWISS-MODEL server.

The structure of the *BocGTR1s* deduced amino acid sequence was analyzed. The results showed that the molecular weights of BocGTR1a,b, and c were 70.44, 68.13, and 68.2 KDa, respectively. The isoelectric point value was 8.71, 8.94 and 8.86; the evaluation of total average hydrophilicity (GRAVY) was as follows: 0.238, 0.209, and 0.253, which indicated that the 3 BocGTR1s are hydrophobic proteins. The hydrophobic part was larger than the hydrophilic part according to the hydrophobicity of deduced protein analysis by ProtScale software (Figure 2A), which verified the prediction above. All three proteins contained a conserved domain of PTR2 (Pfam: PF00854) (Figure 1C), belonging to the proton-dependent oligopeptide transporter (PTR) family.

The membrane-spanning regions of BocGTR1s and its homologous protein AtGTR1 were analyzed by TMpred software (Figure 2B). The location and number of trans-membrane proteins of the three BocGTR1s were similar to those in AtGTR1 in *Arabidopsis thaliana*, suggesting that BocGTR1s and AtGTR1 were very similar in protein structure and may have similar functions related to glucosinolate transport. Signal P 4.1 was used to predict the signal peptide. BocGTR1a, BocGTR1b, and BocGTR1c had no obvious signal peptides and are non-secretory proteins (Figure 2C). BocGTR1b and BocGTR1c proteins contain coiled structures, while BocGTR1a proteins has no coiled structures (Figure 2D).

The secondary structure of the BocGTR1a protein was composed of 42.43% alpha helices, 16.88% extended strands, 32.33% random coils, and 8.36% beta turns (Figure 2E). BocGTR1b and BocGTR1c had similar secondary structures with BocGTR1a. These results showed that changes in the amino acid sequence had no effect on the secondary structure of these proteins, but only on the ratio of different structures. The three-dimensional structures were constructed using SWISS MODEL software. BocGTR1a, BocGTR1b, and BocGTR1c have similar three-dimensional structures (Figure 2F), which indicated that these three proteins may have similar functions.

### 2.3. Phylogenetic Analysis of Three BocGTR1 Proteins

**Figure 3:**
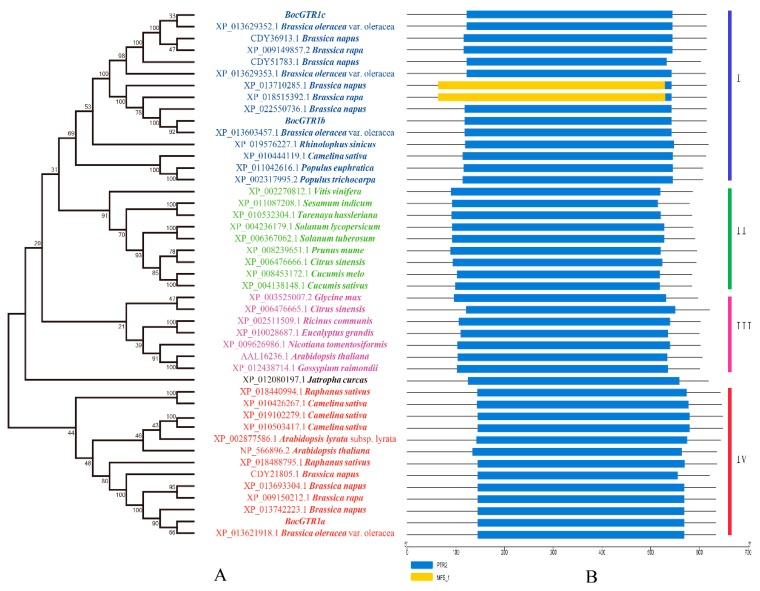
Phylogenetic tree and conserved domain of BocGTR1s with 41 GTR homology proteins from plants. (**A**) This phylogenetic tree was constructed by the neighbor-joining method. Numbers on the tree represent confidence values from the bootstrap test for 1000 replicates. (**B**) The conserved domain of BocGTR1s with 41 GTR homology proteins from plants.

The three BocGTR1s have high similarities (>80%) with the NRT1/PTR-like proteins from cruciferous plants, such as *B. oleracea*, *B. napus*, and *Raphanus sativus*, and a similarity of 60%~80% with the NRT1/PTR (NPF) family members from *Camelina sativa*, *Citrus sinensis*, and *Glycine max*. Three BocGTR1s and 41 homologous proteins from 25 plants were analysed by phylogenetic tree analysis. BocGTR1c was first clustered with three predicted NPF proteins, XP_013629352.1, CDY36913.1, and XP_009149857.2, from *B. oleracea*, *B. napus* and *B. rapa*, respectively, and BocGTR1b were first clustered with another three predicted NPF proteins from *B. oleracea*, *B. napus*, and *B. rapa*, and both BoGTR1b and BoGTR1c belonged to clade I (Figure 3A). BocGTR1a was also first clustered with three predicted NPF proteins XP_013621918.1, XP_013742223.1, and XP_009150212.1 from *B. oleracea*, *B. napus* and *B. rapa*, respectively, in the fourth branch, and these proteins were very far away from clade I (Figure 3A). In addition, several predicted NRT1/PTR proteins from *V. vinifera*, *S. indicum*, *T. hassleria*, and others, were clustered in the second branch. Several predicted NPF proteins from *R. communis*, *G. max*, and *C. sinensis* were clustered in the third branch (Figure 3A). The protein domain was predicted by Pfam and SMART, and the results indicated that every homologous sequence contained the PTR2 domain, belonging to the NPF gene family (Figure 3B).

### 2.4. Expression Profile of BocGTR1s in Different Tissues

**Figure 4:**
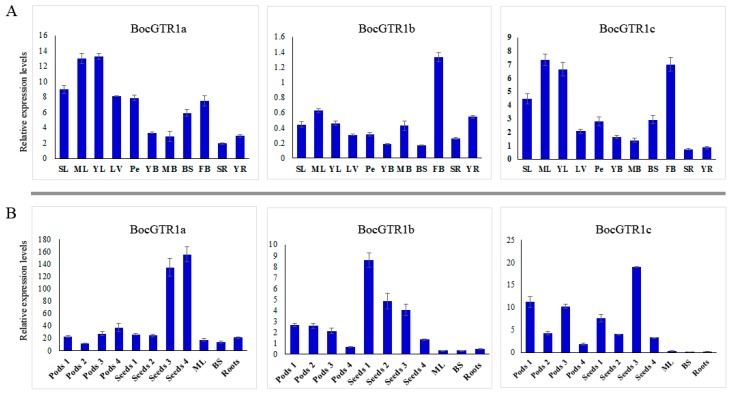
Expression profile of BocGTR1s in different tissues and at different developmental stages. (**A**) The expression patterns of BocGTR1s in different tissues when plants were fully grown, with inflorescences as high as the apical leaves. (**B**) The expression patterns of BocGTR1s in different tissues at the stage of pod development. Sample identifiers: senescent leaf (SL), mature leaf (ML), young leaf (YL), leaf vein (LV), petiole (Pe), young bolting stem flesh (YB), middle bolting stem flesh (MB), bolting stem skin (BS), flower buds (FB), senescent roots (SR), and young roots (YR). Triplicate quantitative PCR experiments were performed for each sample. SD is for the error bars.

The expression patterns of the three BocGTR1s in different tissues of Chinese kale were preliminarily analyzed by qRT-PCR using fully grown plants (Figure S1A). The expression levels of the three BocGTR1s in leaves and buds were higher than those in other tissues, such as leaf veins, petioles, young bolting stem flesh, middle-aged bolting stem flesh, bolting stem skin, and roots. Generally, the expression levels of BocGTR1a and BocGTR1c were higher than those of BocGTR1b in all examined tissues (Figure 4A). This finding indicated that the function of BocGTR1a and BocGTR1c may be more important than BocGTR1b in the role of glucosinolate transport or accumulation.

To identify the role of BocGTR1s in seeds, gene expression patterns were analyzed in the silique coat and seeds of mature siliques (Figure S1B,C). In general, the expression levels of the three genes in silique coats and seeds were higher than the other examined tissues, such as mature leaves, middle-aged bolting stem skins and roots, indicating that, at this stage, the three BocGTR1s were more important in siliques than in other tissues. The expression levels of BocGTR1a were much higher in silique coats and seeds at stage 4 than at the early stage (Figure 4B). However, the expression levels of BocGTR1a were much higher in silique coats and seeds at the early stage than those at the late stage (Figure 4B). BocGTR1c had higher expression levels in silique coats at the early stage, with an expression peak in seeds at stage 3 (Figure 4B). These results indicated that BocGTR1a may play more important roles in siliques at the late stage than at the early stage, but BocGTR1b was more important in siliques at the early stage. In addition, the relative expression levels of BocGTR1a and BocGTR1c were higher than those of BocGTR1b in siliques at different stages and all other examined tissues (Figure 4B).

### 2.5. Expression Profile of BocGTR1s under Different Stress Treatments

**Figure 5:**
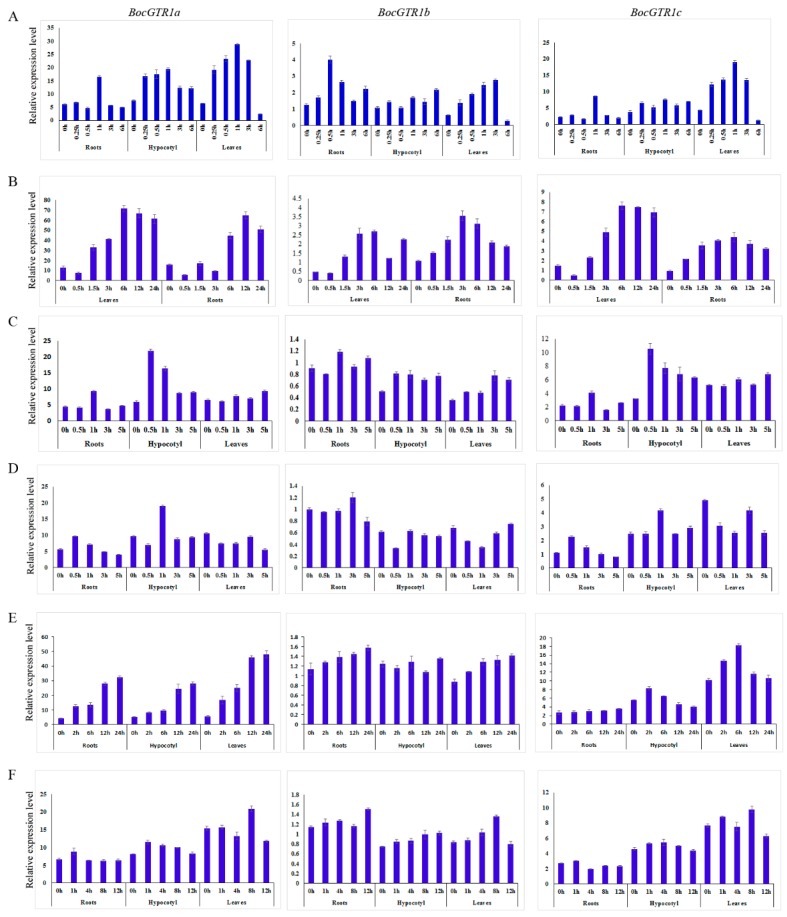
Expression profile of BocGTR1s under different treatments. (**A**) The expression patterns of BocGTR1s in response to wounding. (**B**) The expression patterns of BocGTR1s in response to heat stress. (**C**) The expression patterns of BocGTR1s response to methyl jasmonate. (**D**) The expression patterns of BocGTR1s in response to salicylic acid. (**E**) The expression patterns of BocGTR1s in response to salt (MgCl_2_) stress. (**F**) The expression patterns of BocGTR1s in response to salt (NaCl) stress.

The expression patterns of BocGTR1s in response to injury, heat stress, treatment with methyl jasmonate, salicylic acid, MgCl_2_ and NaCl were analyzed. The expression of BocGTR1a was induced by wounding at 0.25 hours of treatment, peaking after 1 h of treatment and then declining in the roots, hypocotyl, and leaves (Figure 5A). Similarly, the expression levels of BocGTR1b and BocGTR1c were also induced by wounding stress in three examined tissues (Figure 5A). All three BocGTR1s were upregulated in roots and leaves after 3 hours of heat stress by incubation at 40 °C (Figure 5B).

After treatment with 100 µM salicylic acid, the expression levels of BocGTR1a and BocGTR1c increased first and then decreased in roots and hypocotyls, but not in leaves (Figure 5C). The expression of BocGTR1b was not clearly upregulated by salicylic acid treatment (Figure 5C).

The expression of BocGTR1a and BocGTR1c was slightly induced by 100 µM methyl jasmonate treatment in roots and hypocotyls, but not in leaves (Figure 5D). The expression of BocGTR1b was not clearly upregulated by methyl jasmonate (Figure 5D).

The expression of BocGTR1a was increased after 2 h of 10 mM MgCl_2_ treatment until 24 h in roots, hypocotyls, and leaves. After treatment with MgCl_2_, the expression of BocGTR1c in hypocotyls and leaves increased first and then decreased, but it did not change significantly in roots (Figure 5E). The expression levels of BocGTR1b were not induced by MgCl_2_ treatment in any of the examined tissues (Figure 5E). After treatment with 100 mM NaCl, the expression levels of *BocGTR1a* and *BocGTR1c* increased first and then decreased in roots and hypocotyls, but showed no regular expression pattern in leaves (Figure 5F). The expression patterns of *BocGTR1b* was slightly upregulated by NaCl treatment in three tissues (Figure 5F).

### 2.6. BocGTR1a RNAi Transgenic Chinese Kale Plants Exhibited Altered Glucosinolate Levels in Roots and Leaves

**Figure 6:**
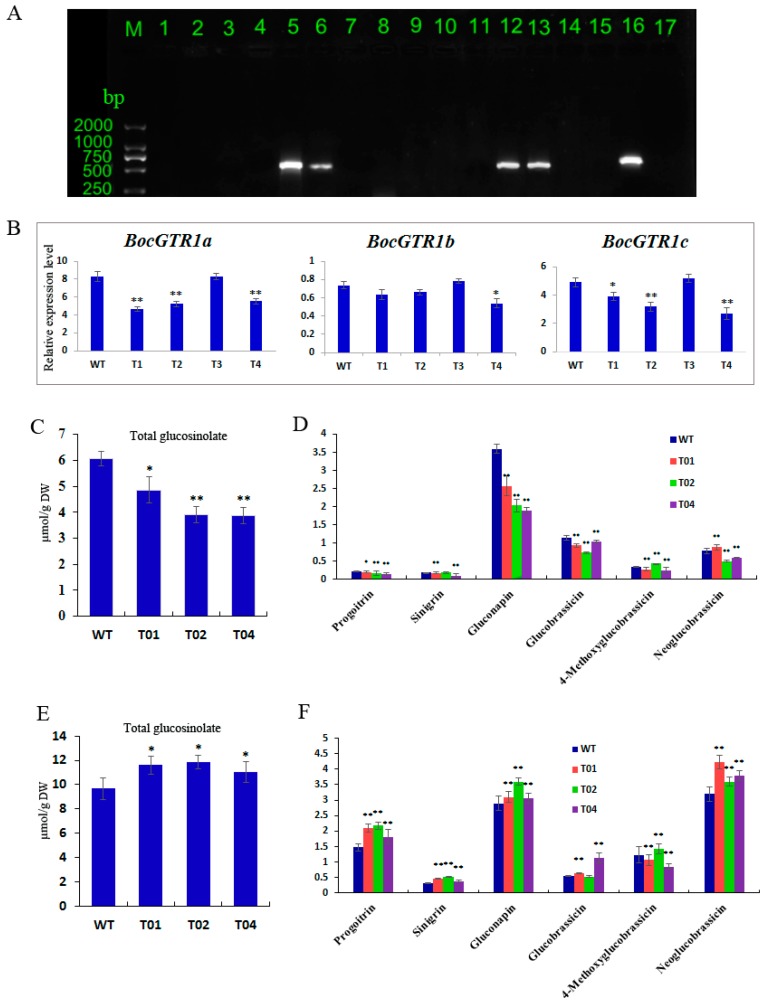
Gene function analysis of BocGTR1a using RNAi technology. (**A**) Detection of PPT-resistant plants by PCR. Lines 1-14: PPT-resistant plant; 15: Wild-type plants; 16: Positive control of RNAi-GTR1a plasmid; 17, Blank control, H_2_O; M:DL2000 Marker. (**B**) Analysis of the expression patterns of 3 BocGTR1s in the leaves of transgene plants and wild-type plants. (**C**) The contents of total glucosinolates in leaves of RNAi transgene plants. (**D**) The contents of different types of glucosinolates in the leaves of RNAi transgene plants. (**E**) The contents of total glucosinolates in roots of RNAi transgene plants. (**F**) The contents of different types of glucosinolates in the roots of RNAi transgene plants. WT: wild-type plants; T01, T02, T03, and T04: RNAi transgene plants; ** represents *p* < 0.01, * represents *p* < 0.05.

To analyse the function of the BocGTR1a gene, the RNAi vector of BocGTR1a was used for transformation. Approximately 3500 Chinese kale explants were used to carry out the *Agrobacterium*-mediated transformation, and 11 PPT-resistant lines were obtained following regeneration. When these lines were analyzed by PCR using the oligonucleotide primers specific for the bar gene sequence, only four of them showed the expected amplification product (Figure 6A, lanes 5, 6, 12, and 13). No amplification signal was observed for the untransformed control line (Figure 6A lane 17). The results preliminarily demonstrated that the BocGTR1a RNAi gene had been integrated into the genome of the transgenic lines. These transgenic plants (named T1, T2, T3, and T4) were propagated, rooted, and transferred into the greenhouse. The expression of BocGTR1s in transgenic plants was analysed by qRT-PCR using RNA extracted from leaves of four PCR-positive transgenic plants as a template. The expression levels of BocGTR1a were decreased significantly in the RNAi plants T1, T2, and T4 compared to wild-type plants (Figure 6A), suggesting that the expression of BocGTR1a was knocked down in RNAi-BocGTR1a plants. However, the expression of BocGTR1a in T3 plants was not reduced (Figure 6B), which indicated that BocGTR1a was not successfully knocked down in T3 plants. Therefore, the RNAi plants T1, T2, and T4 were chosen for further study. The expression patterns of BocGTR1b and BocGTR1c were analyzed in RNAi plants. Interestingly, the expression levels of BocGTR1c were also decreased significantly in the RNAi plants T1, T2, and T4 compared to wild-type plants (Figure 6B), as a result of the high similarity between the cDNA sequence of BocGTR1a and BocGTR1c. However, the expression levels of BocGTR1b were not significantly decreased in any RNAi plants, which may be because BocGTR1b was expressed in Chinese kale plants at a very low level.

To analyse the function of BocGTR1a involved in glucosinolate transport or reaccumulation in Chinese kale, the contents of different glucosinolates were determined in the RNAi and wild-type plans. In this experiment, the glucosinolate content of the sixth leaf and roots from RNAi (T1, T2, and T4) and wild-type plants were analyzed by high-performance liquid chromatography (HPLC). Two biological replicates were conducted, and similar results were obtained. Three technical replicates were performed for each biological replicate. When the BocGTR1a gene were knocked down in RNAi plants, the levels of total glucosinolates were significantly decreased in the leaves of RNAi plants compared with the wild-type plants (Figure 6C), and the content of gluconapin, glucobrassicin and progoitrin were also significantly decreased in the leaves of three RNAi plants (Figure 6D). In addition, total glucosinolates in roots were increased significantly in three RNAi plants (Figure 6E), and the contents of progoitrin, sinigrin, and neoglucobrassicin were also increased in RNAi plants compared with the WT plants (Figure 6F). However, some of the glucosinolate compounds, including sinigrin and Glucobrassicin, were not altered in all the three RNAi plants, which indicated that BocGTR1a may have different roles in the transport of different glucosinolate compounds.

## 3. Discussion

As sessile organisms, plants depend on their vast array of chemical weapons for defense against herbivores and pathogens, and these defense compounds accumulate to their highest levels in tissues that are most likely to be attacked [5,21]. As a group of defense compounds, glucosinolates comprise a large family of over 130 plant amino acid-derived secondary metabolites mainly found in the Brassicaceae [22]. Glucosinolates are synthesized in every part of plants except seeds and are then transported to their storage positions, which may include seeds and other plant parts [9,10]. GTR1 and GTR2 play important roles in the glucosinolate transport process and control the loading of glucosinolates from the apoplasm into the phloem in *Arabidopsis* [5]. In the present study, three glucosinolate transporter genes, BocGTR1s, were cloned from Chinese kale. They shared the same PTR transmembrane domain as AtGTR1 and belonged to the nitrate/oligopeptide transporter family (NTR/PTR). In *Arabidopsis*, there is only one GTR1 gene with a DNA length of 2798 bp [5], which is exactly the same as the DNA sequence length of BocGTR1a. The largest ORFs of BocGTR1a, b and c were also very close to AtGTR1, which indicated that the GTR homologies cloned in Chinese kale were correct. Based on the conserved syntenic block analysis between the genomes of *Brassica* plants and *A. thaliana*, the hypothesis of an ancestral karyotype was proposed, and comparative physical mapping studies confirmed genome triplication in *B. rapa* from *Arabidopsis* [23,24]. Through BLAST analysis, more than one GTR1 orthologs was identified in *B. oleracea*, *B. napus*, and *Brassica rapa*, and most of these GTR1s were clustered together (Figure 3A). These results indicated that there were several GTR homologous proteins in Chinese kale or other *Brassica* plants, which maintained the glucosinolate transport function together, and there were key GTR proteins controlling glucosinolate transport in different tissues.

Understanding the expression pattern analysis of genes is helpful in understanding their roles in plant physiological processes. Three *BocGTR1s* had higher expression levels in leaves and buds than other examined tissues, and the expression levels of BocGTR1a and BocGTR1c were much higher than those of BocGTR1b in all examined tissues of full-grown plants (Figure 5A). These results indicated that BocGTR1a and BocGTR1c may be the key glucosinolate transporter genes in Chinese kale, and they may play roles in leaves and buds in full-grown plants. In addition, the expression levels of the three BocGTR1 genes in silique coats and seeds were higher than in mature leaves, bolting stem skins and roots, and three BocGTRs had different expression peaks at different stages of seed maturation (Figure 5B). These results suggested that at this stage, the three BocGTR1s were more important in siliques than in other tissues, and BocGTR1a may play more important roles in siliques at the late stage than at the early stage, but BocGTR1b was more important in siliques at the early stage than at the late stage. These expression patterns were consistent with the present results that glucosinolates were synthesized in other plant parts and eventually accumulated in seeds by GTR protein transport [5,25].

The biosynthesis and accumulation of glucosinolate in different tissues can be modified by abiotic and biotic stress factors [26,27,28]. To our knowledge, very few studies have reported on the expression levels of the GTR gene in response to various abiotic stresses under different treatments. Recently, it was reported that stress caused by wounding induces the accumulation of important bioactive compounds in *Brassica* plants, such as glucosinolates [29,30]. Many genes involved in both aliphatic and indolyl glucosinolate pathways were induced by wounding [30]. In the present study, all three transport-related genes were strongly induced by wounding stress. In addition, except for NaCl treatment, the expression of the three *BocGTR1s* was upregulated by wounding, heat stress, and treatment with methyl jasmonate, salicylic acid, and MgCl_2_, which indicates that the transport and accumulation of glucosinolate may be affected by most abiotic stresses and hormones involved in response to biotic stresses. However, what mediates the cross talk between the accumulation of glucosinolate and abiotic stress requires further study.

Because of the high sequence similarities (>95%) among the three BocGTR1s, we simultaneously knocked down the expression of the three BocGTR1s using one RNAi vector. As expected, the expression of BocGTR1a and BocGTR1c in the leaves of T_0_ transgenic plants was significantly knocked down, but expression of BocGTR1b was not reduced significantly, which may be because of the very low expression level of BocGTR1b in Chinese kale plants. The total glucosinolate content in the sixth leaves of T_0_ transgenic plants was significantly lower than that of the wild-type control, while the total glucosinolate content in roots was significantly higher than that of the negative control. It was presumed that glucosinolates are mainly synthesized in buds, leaves and roots during plant growth and development and then transported and distributed to various tissues based on proximity [24,31,32]. We speculated that, due to the reduction of BocGTR1a and BocGTR1c expression levels, resulting in the observed low concentration of glucosinolates in mature leaves (sixth leaves) of RNAi plants (Figure 6C). As a result of knocking down BocGTR1a and BocGTR1c, the glucosinolate synthesized in roots could not be transported to the stems and leaves and resulted in the high accumulation of glucosinolate in roots (Figure 6E). The difference between the relative glucosinolate content in the phloem and the leaves suggests that there is a selection for specific glucosinolates to be loaded into the phloem [11]. After partial silencing of the BocGTR1a and BocGTR1c genes, only some glucosinolates had altered levels, suggesting that BocGTR1a and BocGTR1c participate in the transport of glucosinolate with a selection for specific glucosinolates. Recently, one out of seven GTRs from *B. rapa* and four out of twelve GTRs from *B. juncea* were mutated and they reduced glucosinolate levels in seeds by 60%–70% in these two different *Brassica* species [12]. These genes were selected for gene mutation because of their high relative expression in seeds [12]. In the present study, since *BocGTR1a* and *BocGTR1c* have similar sequence, structure and expression profile, they may be redundant. *BocGTR1b* showed very low expression levels, which suggested that BocGTR1b may have specific functions. However, the exact roles of three BocGTR1s involved in glucosinolate transport need to be proved by more experiments, such as heterologous expression and gene mutation.

## 4. Materials and Methods

### 4.1. Plant Materials, Growth Conditions and Stress Treatments

A local variety of Chinese kale was cultured in the experimental field of South China Agricultural University (Guangzhou, China). The style of field management was identical to general production practices specific to Chinese kale. The plants were cultured in the natural field soil, with artificial irrigation, at a temperature of 20~30 °C. Different types of samples for RNA extraction and sequencing were harvested free of any insects and mechanical damage when the plants were fully grown (about 50 d after seeds imbibition), with inflorescences as high as the apical leaves [33]. For methyl jasmonate treatment, the plants were grown in pots with regular light/dark (16 h/8 h) cycles for 30 days, and the plants were sprayed with 100 µM methyl jasmonate. The leaves, hypocotyl, and roots were sampled after treatment at 0 h, 30 min, 1, 3, and 5 hours. For the salicylic acid treatment, 30-day-old plants were sprayed with 100 µM salicylic acid. The leaves, hypocotyls and roots were sampled after treatment at 0 h, 30 min, 1, 3 and 5 hours. For salt stress, the roots of 30-day-old plants were watered with 100 mM NaCl or 10 mM MgCl_2_. The leaves, hypocotyls and roots were sampled after treatment at 0, 1, 4, 8, 12, and 24 h. For wounding stress, the leaves of 30-day-old plants were stuck with a needle, producing 16 holes. The leaves, hypocotyls, and roots were sampled after treatment at 0, 0.25, 0.5, 1, 3, and 6 h. For heat stress, 30-day-old plants (initially planted at 25 °C) were transferred to an incubator and held at 40 °C. The leaves and roots were sampled after treatment at 0, 0.5, 1, 3, 6, 12, and 24 h. The roots were washed with sterile water and dried with sterile paper. There were at least six plants per conditions. All these samples were immediately frozen in liquid nitrogen and stored at −80 °C until further analysis.

### 4.2. Molecular Cloning of Three BocGTR1s

Genomic DNA was extracted from young leaves by CTAB following the method of Murray et al. [34]. All RNA extractions, PCR, and cDNA synthesis were performed, as previously described [35]. To obtain the full-length cDNA and DNA sequence, PCR was performed using cDNA and genomic DNA of Chinese kale as templates, respectively. The full-length cDNA and DNA were amplified with the primer pairs P1, P2, and P3 listed in Figure S1. The amplified PCR products were purified, cloned, and then sequenced.

### 4.3. Sequence Analysis

NCBI BLAST was used for retrieval and alignment of the nucleotide sequence. We used DNAMAN software for gene multiple sequence alignment. Gene structure was analysed using GSDS2.0 software (http://gsds.cbi.pku.edu.cn/). Protein physical and chemical properties were analyzed using the ProtParam tool (https://web.expasy.org/protparam/). Protein hydrophilicity was analyzed using ProtScale software (https://web.expasy.org/protscale/). Signal peptides were predicted by SignalP 4.1 Server (http://www.cbs.dtu.dk/services/SignalP/). Protein trans-membrane regions were predicted by TMpred Server software (http://www.ch.embnet.org/software/TMPRED_form.html). The secondary structures of the proteins were predicted by SOPMA (http://bioinf.cs.ucl.ac.uk/psipred/).

SWISS-MODEL software (https://swissmodel.expasy.org/interactive) was used to predict the tertiary structure of the proteins. The subcellular localization of the proteins was predicted by CELL (http://cello.life.nctu.edu.tw/). The phylogenetic tree was constructed using Mega7.0 software [36].

### 4.4. Expression Analysis by qRT-PCR

Quantitative real-time RT-PCR was carried out using a SYBR Green I Mix (TaKaRa, Dalian, China) as previously described [35]. Gene-specific and *β-actin*-specific primers were designed for the three BocGTR1s and *β-actin* (Supplementary Table S1). Triplicate technical replicates in quantitative PCR experiments were performed for each sample, and the expression values obtained were normalized against *β-actin*. Analysis of the relative gene expression data was conducted using the 2^−∆∆^Ct method [37].

### 4.5. Vector Construction and Transformation of the BoGTR1a RNAi gene

The BoGTR1a RNAi vector was constructed as described previously [38]. Briefly, a 227-bp forward DNA fragment of the BocGTR1a cDNA was amplified by PCR (primer pair listed in Supplementary Table S1), cut with *Swa*I and *Nco*I, and transferred into the vector pFGC5941. The reverse DNA fragment of the *BocGTR1a* cDNA was amplified by PCR (primer pair listed in Supplementary Table S1), cut with *BamH*I and *Sma*I, and transferred into the vector pFGC5941. The binary vectors were introduced into the *Agrobacterium tumefaciens* strain EHA105.

The BocGTR1a RNAi gene was introduced into hypocotyls of Chinese kale ‘No.5’ using the *Agrobacterium tumefaciens*-mediated method [39]. To verify the presence of the bar-coding sequence in the RNAi transgenic plant genome, PCR was carried out using pBar1 and pBar2 (Supplementary Table S1) [38]. The PCR products were detected by electrophoresis in a 1.5% agarose gel. For BocGTRan expression pattern analysis in RNAi transgenic plants, qRT-PCR was performed as above.

### 4.6. Extraction and Determination of Glucosinolates

The glucosinolate content of leaves and roots was determined using the HPLC method, as described previously [20]. Briefly, glucosinolate was extracted from 0.1 g of lyophilized sample by adding 4 mL of 70% methanol in a 75 °C water bath for 20 min. Then, barium acetate was added, followed by centrifugation. Five milliliters of extraction solution and 500 μL of 0.5 mg/mL sulfatase solution were slowly flowed through a homemade column. The reaction was carried out at 28 °C for 12 h and eluted with 2 mL ultra-pure water. The mobile phase A (ultrapure water) and B (acetonitrile) change gradient was: 0–32 min, 0%–20% acetonitrile; 33–38 min, 20% acetonitrile; and 39–40 min, 20%–100% acetonitrile. The detection wavelength was 229 nm, the flow rate was 1 mL/min, and the column temperature was 30 °C. Each sample was duplicated in parallel, using 100 μL of 5 mg/mL 2-propenylglucoside as an internal standard.

## 5. Conclusions

In this study, three glucosinolate transporter-related genes were cloned from Chinese kale. The amino acids of the three genes have more than 81% homology with AtGTR1 from *Arabidopsis thaliana*. The trans-membrane structures and functional domains of the three genes were identical to those of *Arabidopsis* AtGTR1, suggesting that they may have similar functions to *Arabidopsis* AtGTR1. The expression levels of BocGTR1a and BocGTR1c were much higher than those of BocGTR1b in various tissues, especially in leaves and buds. Magnesium chloride treatment, wounding and heat stress could significantly induce the expression of *BocGTR1s*. The expression levels of BocGTR1a and BocGTR1c in leaves were significantly silenced by RNAi interference. The silencing of BocGTR1a caused a reduction in total glucosinolate levels in leaves and an increase in roots. In addition, we found that BocGTR1a and BocGTR1c may participate in export with a selection for specific glucosinolates. BocGTR1a and BocGTR1c may be the key genes involved in glucosinolate transport and accumulation in different tissues of Chinese kale.

## Supplementary Materials

The following are available online at https://www.mdpi.com/2073-4425/10/3/202/s1, Figure S1: Different tissues used in RNA-seq analysis: (**A**) The plants were full-grown, with inflorescences as high as the apical leaves. Sample identifiers: senescent leaves (SL), mature leaves (ML), young leaves (YL), leaf veins (LV), petioles (Pe), young bolting stem flesh (YB), middle-aged bolting stem flesh (MB), bolting stem skin (BS), flower buds (FB), senescence roots (SR), and young roots (YR). (**B**) The plants were at the stage of seed maturation. (**C**) The siliques used in the qPCR. DAF: Days after flower. Table S1: Primers used in the present study.
