# Supplementary material for: Molecular Cloning and Characterization of Three Glucosinolate Transporter (GTR) Genes from Chinese Kale
Source: Genes (Basel). 2019 Mar 8;10(3):202. doi: 10.3390/genes10030202 (PMC6471314; doi:10.3390/genes10030202)
Supplement: Supplementary file 1 [file genes-10-00202-s001.pdf]

Supplementary

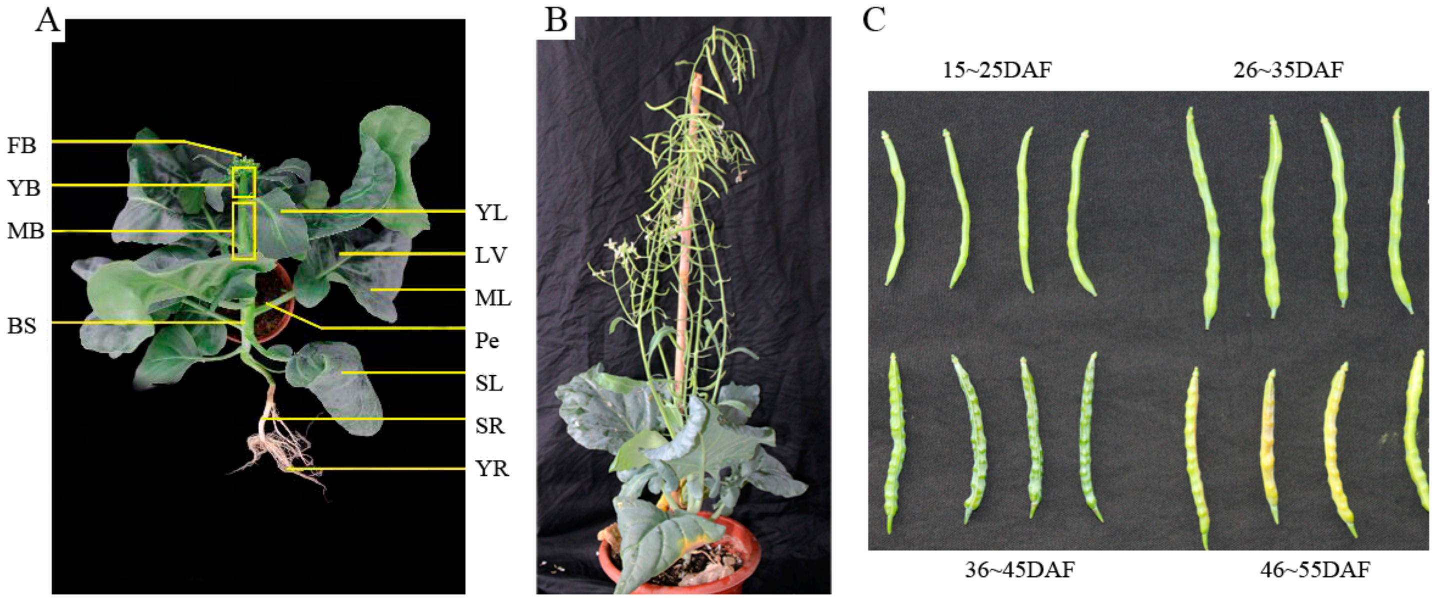

Figure S1. Different tissues used in RNA-seq analysis: **(A)** The plants were full-grown, with inflorescences as high as the apical leaves. Sample identifiers: senescent leaves (SL), mature leaves (ML), young leaves (YL), leaf veins (LV), petioles (Pe), young bolting stem flesh (YB), middle-aged bolting stem flesh (MB), bolting stem skin (BS), flower buds (FB), senescence roots (SR), and young roots (YR). **(B)** The plants were at the stage of seed maturation. **(C)** The siliques used in the qPCR. DAF: Days after flower.

Table S1. Primers used in the present study.

**No.**	Gene name	Specific primers		Product size(bp)	Purpose
		Forward (5′–3′)	Reverse (5′–3′)		
P1	BocGTR1a	TAAAAAGACGATAGAGAGGA	GGAAGTACAACAACAAAGAT	1905	For cDNA clone
P2	BocGTR1b	GGGAGAGAAGAGACAAG	ACAACAAAGAGGGAGAC	1848	For cDNA clone
P3	BocGTR1c	AGACGGAGAGAAGATAAAAGA	ACGGAGTACAGTACAACAACA	1848	For cDNA clone
P4	BocGTR1a	GCTTACACCCTCCCTCTCTCTC	TTATTGCCTTATCTCCCATTCC	2798	For DNA clone
P5	BocGTR1b	ATAGGGAGAGAAGAGACAAG	GTTTAGAAAGATGAAGTGGT	2365	For DNA clone
P6	BocGTR1c	AGACGGAGAGAAGATAAAAGA	ACGGAGTACAGTACAACAACA	2605	For DNA clone
P7	BocGTR1a	GGGGTACCATGAAGAGCAGAGTCGTCCT	TCCCCCGGGCAGAGTTCTTGTCTTGTT	1905	Subcellular localization clone
P8	BocGTR1b	GCTCTAGAATGGAGAGAAAGCCCTTTGA	GGGGTACCACATAGCTCTTGTCTTGTT	1848	Subcellular localization clone
P9	BocGTR1c	ACGCGTCGACATGGAAAGAAAGCCTTTTGA	TCCCCCGGGCCGAGTTCTTCTCTTGCT	1848	Subcellular localization clone
P10	BocGTR1a	TTCATCATAATCTACGACCG	GAGACCAATAAACTCAACAC	127	For qPCR
P11	BocGTR1b	AGTGAAAGCCTCGGGTAG	CGGAGTATTTGAGTGTAG	152	For qPCR
P12	BocGTR1c	TCATTTTCTTCGCTGGTCA	TCGGTGTATTTTAGGGTCA	187	For qPCR
P13	Actin	GAGGCTCCTCTTAACCCAAA	CAGAATCAAGCACAATACCG	148	For qPCR
P14	BocGTR1a	ttacatttacaattaccatggGGCCGTGTAACTTGGCCTT	tcgattgggcgcgccccatggGTGACCAGCAAAGAAAATGACGC	224	For Justice fragment of RNAi clone
P15	BocGTR1a	CGCGGATCCTGGTGGGTGCTGGTGGAATCA	TCCCCCGGGGGCTTTCACTTTCACATACAGTTT	224	For Antisense fragment of RNAi clone
P16	Bar	CCCTTATCTGGGAACTACTCACACA	TTCATTTCATTTGGAGAGGACACGC	669	For Bar clone
